# Subphrenic abscess through esophageal leakage after laparoscopic initiated nissen fundoplication -Case presentation-

**Published:** 2008-04-15

**Authors:** Constantinoiu S., Chiru Fl., Beuran M., Streinu-Cercel A., Cociu Luminita, Constantin A., Lintoiu Beatrice, Ursut B., Hoara P.

**Affiliations:** * General and Esophageal Surgery Clinic, „Sf. Maria” Hospital; ** Clinic of General Surgery, „Floreasca” Emergency Hospital; *** Infectious Diseases Clinic, „ Matei Balş” Infectious Diseases Institute; **** Intensive Care, „Sf. Maria” Hospital

**Keywords:** subphrenic abscess, laparoscopic fundoplication, esophageal leakage, sepsis

## Abstract

A 56-year-old man with a large paraesophageal hiatus hernia, treated in a foreign clinic with a Nissen fundoplication (when a lesion of the gastric fornix during laparoscopic dissection has determined conversion to open technique) is admited 3 weeks after surgery, being diagnosed with an esophageal leekage witch maintains a large subphrenic abscess with sepsis. The patient was cured by draining the leakage, excluding the esophagus by an “à minima” alimentary jejunostomy, under broad spectrum antibiotherapy.

The patient VLP, male, aged 56, was known to have a giant paraesophageal hiatus hernia for almost 20 years (see **fig.1 a, b**; **fig.2 a, b**). Functional signs – relatively severe – consisted of respiratory troubles and anemia with Hb = 8g/dl; associated: 3rd degree obesity for a short constitutional type. He did not present neither gastro-esophageal reflux nor esophagitis.

**Fig. 1:**
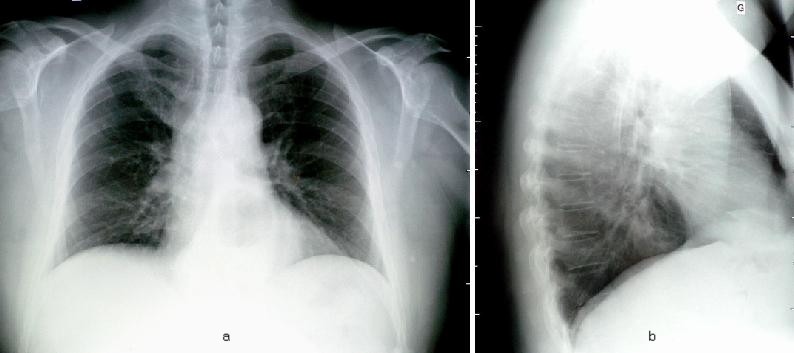
- a - gaseous image superposed to the heart shadow (stomach herniated in the thorax) - b – the same mediastinal gaseous image situated posterior

Although in our clinic we established the preferential indication for open surgery, taking into account the volume and age of hernia, as well as the possibility of the existence of difficult to dissect filamentous adherents, the patient’s option was a laparoscopy approach.

**Fig. 2:**
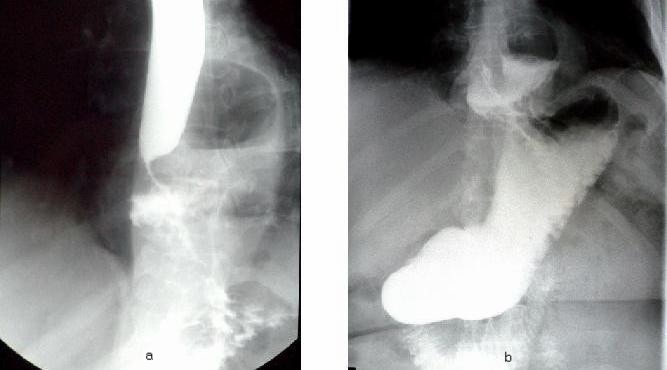
a, b – Rx G-D: large paraesophageal hiatus hernia

On the 11th of January 2008, the patient underwent surgery in a private clinic abroad. Initially, laparoscopy was applied, then converted into open surgery and Nissen fundoplication was performed. The immediate outlook was favourable. After surgery, a moderate and persistent dysphagia for solid substances was present. We don’t have any documents regarding the surgery.

The third week after surgery, the patient returned to his home country; fever appeared along with alteration of his general condition, sepsis and hyperleucocytosis (white cell count = 20 000/mm3). The following investigations were made in Floreasca Emergency Hospital: barium passage, through which we discovered a leakage of the abdominal esophagus (see **fig.3**) and computer tomography which suggested a subphrenic abscess situated between the gastric curve and visceral face of the hepatic left lobe (**fig.4**).

**Fig. 3:**
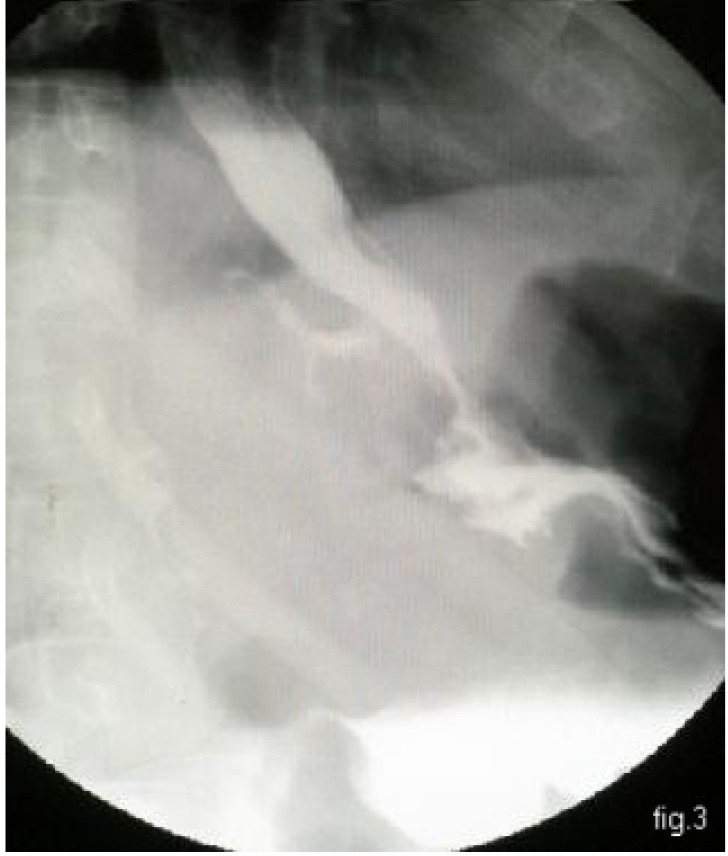
Rx – G-D: the leakage of the abdominal esophagus;

**Fig. 4:**
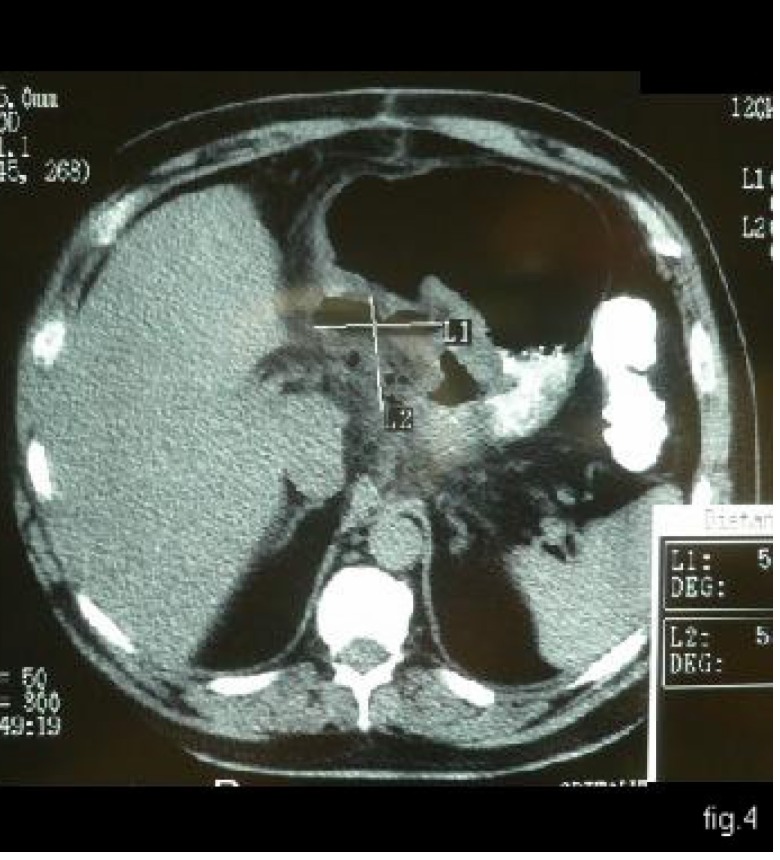
CT – collection with air-fluid level on the little gastric curve

The surgeon was contacted by phone and he confirmed an intraoperative lesion of the gastric fornix during the laparoscopy dissection, sutured after conversion; there were no other lesions. Therefore, the esophageal leakage, with its subsequent subphrenic abscess had no apparent aetiology.

In our clinic we practiced an esophageal-gastrofibroscopy in the surgery room, just before the surgical intervention, with the intubated patient, which confirmed an esophageal leakage at 36 cm away from the dental arcade, with a diameter of 6-8 mm, through which pus flowed spontaneously and at extrinsic compression of the abdomen (**fig.5**).

Surgery continued the same day. A large subphrenic abscess was evacuated (approx. 300ml of creamy pus with bad smell); methylene blue was introduced through the Faucher esogastric tube, with the substance appearing in the cavity of the abscess. As the esophageal wall was infiltrated, inappropriate for a digestive anastomosis, proximity drainage of the esophageal leakage and temporary “à minima” feeding jejunostomy was performed, with a view to exclude the esophagus from the digestive circuit. After surgery, Imipenem (Tienam) 4g/day and Vancomicyne 2g/day were administered; cultures of pus, made afterwards at “Matei Balş” Infectious Diseases Institute, found *Acinetobacter* and *Peptostreptococcus*, germs with sensitivity to the antibiotherapy already initiated.

**Fig. 5:**
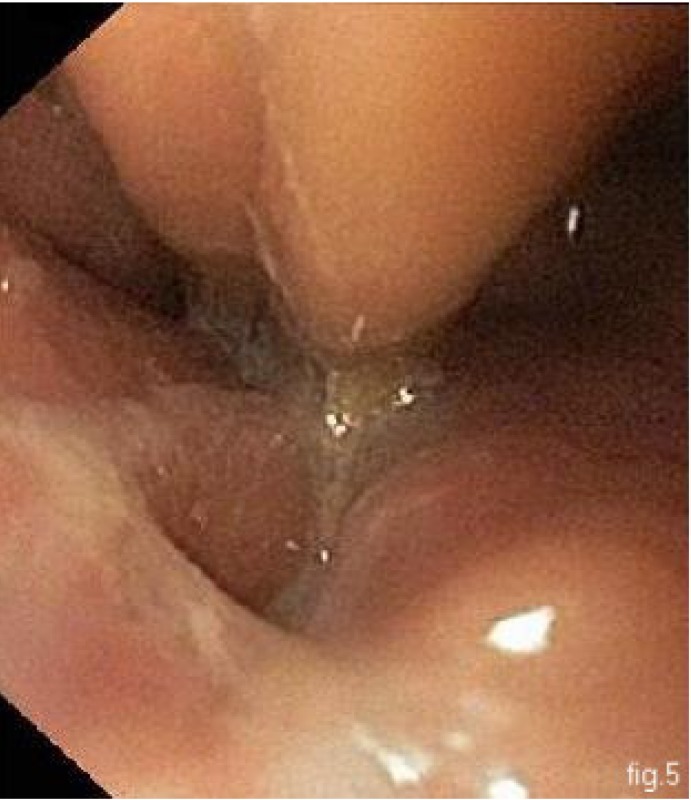
esophago-gastrofibroscopy: esophageal leakage at 36 cm from the dental arcade;

**Fig. 6:**
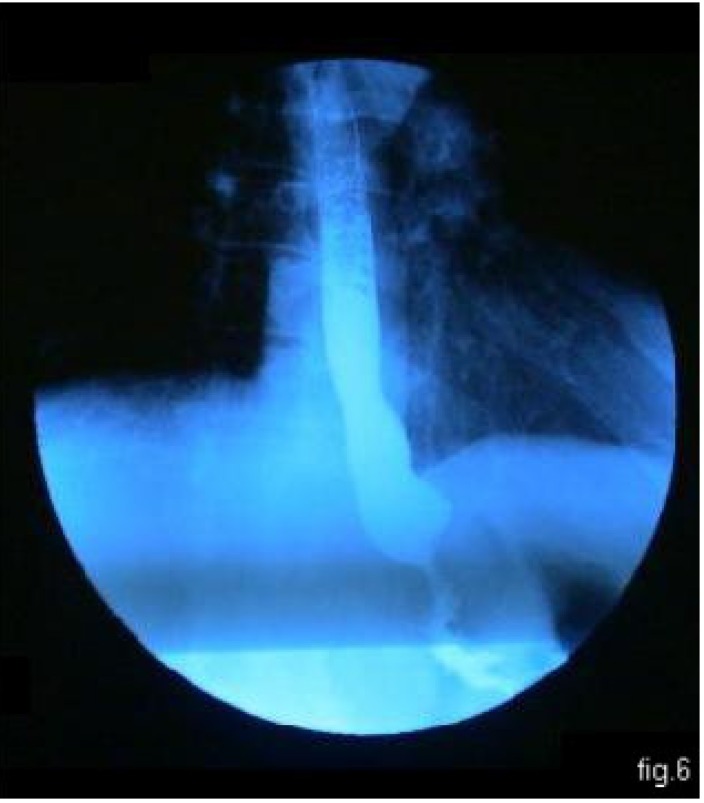
barium passage: normal esophageal passage, without any leakage

After surgery, the outlook was simple. Oral feeding was stopped; feeding through the jejunostomy tube was initiated very early, starting with the 2nd day, with Nutren and Freshubin. The control barium passage, with hydro soluble radio opaque substance, done in the 8th day after surgery, showed a normal esophageal passage, without any leaks (**fig.6**). The patient was discharged in the 11th day 11 days after the surgery. A new radiology control examination made on the 14th day confirmed the healing of the leakage. Thereafter, oral feeding was introduced again; after another 7 days the jejunostomy tube was pulled out

## Discussions

In the case of a large and old hiatus hernia, a difficult dissection is expected due to filamentous adherents. One can try the laparoscopic approach, but the conversion must be very early, before the occurrence of severe lesions. The option of surgery can be the indication of choice. There are possible iatrogenic complex lesions. In our case, “the mirage of the first fornix lesion”, even if discovered and repaired correctly through well indicated conversion, led to missing the second, esophageal, lesion, much more serious, probably an electric injury of the esophageal wall produced with the Hook electrode, detached in the second week of evolution, with the afterward installation of the esophageal leakage and the subphrenic abscess.

The treatment of these serious complications did not tempt the primary approach of the esophageal leakage – an illusory goal – technical and tactical. The surgical strategy pursued two objectives, after the evacuation of the abscess: the proximity drainage of the leakage, associated with excluding the esophagus from the alimentary transit. This way we obtained the secondary healing of the leakage, with a very good result, including a functional one.
